# Analysis of current and future bioclimatic suitability for C. arabica production in Ethiopia

**DOI:** 10.1371/journal.pone.0310945

**Published:** 2024-10-23

**Authors:** Asnake Adane

**Affiliations:** Department of Geography and Environmental, Wollo University, Ethiopia; National Cheng Kung University, TAIWAN

## Abstract

The coffee sector in Ethiopia is the livelihood of more than 20% of the population and accounts more than 25% of the country’s foreign exchange earnings. Climate change is expected to affect the climatic suitability of coffee in Ethiopia, and this would have implications for global coffee output, the national economy, and farmers’ livelihoods in Ethiopia. The objective of this paper is to assess the current and future impacts of climate change on bioclimatic suitability to C.arbica production in Ethiopia. Based on the current distribution of coffee production areas and climate change predictions from HadGEM2 and CCSM2 models and using the Maximum Entropy (MaxEnt) bioclimatic modeling approach, future changes in climatic suitability for C. arabica were predicted. Coffee production sites in Ethiopia were geo-referenced and used as input in the MAXENT model. The findings indicated that climate change will increase the suitable growing area for coffee by about 44.2% and 30.37% under HadGEM2 and CCSM2 models, respectively, by 2080 in Ethiopia. The study also revealed a westward and northwestward shift in the climatic suitability to C. arabica production in Ethiopia. This indicates that the suitability of some areas will continue with some adaptation practice, whilst others currently suitable will be unsuitable, yet others that are unsuitable will be suitable for arabica coffee production. These findings are intended to support stakeholders in the coffee sector in developing strategies for reducing the vulnerability of coffee production to climate change. Site-specific strategies should be developed to build a more climate resilient coffee livelihood in the changing climate.

## Introduction

At a global level, many coffee-producing regions spatially coincide with areas considered as biodiversity "hotspots" [1]. The East Africa Biodiversity Hotspot is anticipated to experience the brunt of climatic change [2]. With this, the distribution and sustainability of coffee production systems have been affected by global climate change [3].

Ethiopia is the origin and diversity of coffee [4], yet is facing the impacts of climate change. It is one of the major coffee-producing countries, and its coffee production has increased in the last 25 years. This is attributed to the expansion of new production areas by changing land use, not increasing yield on existing land (Minten et al., 2018). For example, over the past 10 to 15 years, while production increased by 3600 tonnes/year, annual yield decreased by 18.8kg/ha [5]. This implies an expansion of the coffee production area; notwithstanding, the cause and extent of expansion remains unclear.

Coffee is an important crop in Ethiopia, both ecologically and economically. Coffee agroecosystems provide ecological services like moderating the local climate and reducing soil erosion [6]. In terms of economy, coffee plays a pivotal role in the socio-economy of Ethiopia; it is the livelihood of more than 20% of the economically active population and contributes more than 25% of the country’s foreign exchange earnings in the context of changing climate [2].

Nevertheless, coffee crops are highly sensitive to changes in climate, which can decrease both the quantity and quality of harvests [6,7]. Thus, climate change could severely affect the available growing regions of coffee; cause alteration of climate suitability forcoffee crop and its production intensity, in turn,leads to a change in the spatial range of coffee production [8,9]. Many of the ideal areas of coffee production suffer from increased temperature and variation in rainfall patterns[10,11], and some studies have lamented that growing quality coffee will be impossible by 2080 [10]. Research on how climate change will affect coffee crop suitability have revealed that the places that are appropriate for coffee production will shrink and shift to higher elevations [11]. production The coffee sector faces formidable challenges due to climate change interms of suitability, quality and quantity [12].

However, research on the potential impact of climate change on aspects of coffee has remained far apart in space and time [12]. Particularly, climate change’s current and predicted impacts on coffee in Ethiopia have been understudied [13], and few studies have yielded inconsistent and contradictory findings about the future effects of climate change on the spatial range of coffee production in Ethiopia. For example, Davis et al.[14] reported that the optimal available land for Arabica coffee production in Ethiopia could drastically decrease by 2080 due to climate change. In contrast, Läderach et al. [15] and Perfecto et al. [16] pointed out that climate change would increase the spatial range of coffee production in Ethiopia due to the mountainous nature of the country. This indicates mixed findings on climate change’s effect on coffee production in Ethiopia, highlighting a need for further research. Moreover, majority of studies on current and future impacts of climate change on coffee have been conducted in the Americas; albeit Eastern African countries like Ethiopia’s coffee livelihoods have also substantial contribution to the local,national global economies [17,18] as well as generates ecological services [19]. While many studies attempt to address the effects of the current and past climate changes on Ethiopia’s coffee sector, the predicted impacts of climate change on the spatial range of coffee production in Ethiopia are overlooked [7]. A detailed spatial analysis of current and future coffee production under climate change is important to inform policymakers to devise robust conservation strategies for sustainable coffee livelihoods in Ethiopia [20].

The current study aims to assess Ethiopia’s current and potential climate suitability of C. arabica production areas. The specific objectives are i) to model the distribution of climate suitable to coffee production in Ethiopia based on data on where coffee is grown at present; ii) to compare the difference between the current and the projected climatic suitability as being the modeled impact of climate change on coffee suitability; and iii) discuss the change in areas suitable to coffee production within Ethiopia that may attribute to changes in bioclimatic variables.

## Materials and methods

### Study area

**Fig 1:**
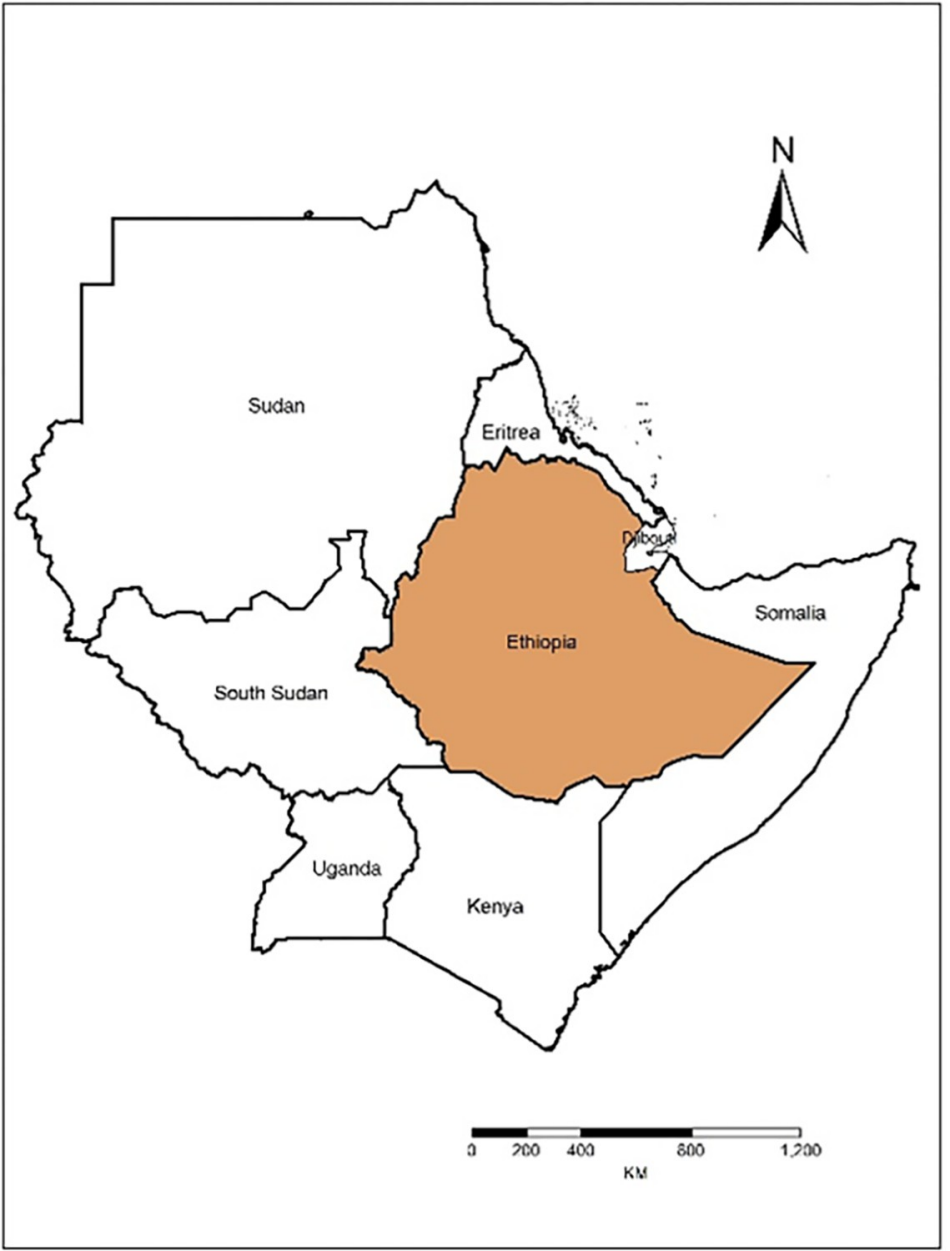
Location map of Ethiopia in the horn of Africa (extracted from https://hub.arcgis.com/datasets/esri::world-countries/explore?location=-1.440657%2C0.000000%2C3.00).

Ethiopia is located in the horn of Africa (Fig 1), as a part of the eastern African highlands. Ethiopia, with an area of 1, 106,000 km^2^, has a highly diversified terrain and mountain environments with high elevations (4,620 m asl) in Seimen mountain and low elevations (<125 m bsl) in the Afar Triangle. About 56% (619,360k km^2^) of the country’s area is highland. The highland part of the country consists of both actual and potential coffee production areas, as climatic suitability for coffee production moves upward to the higher altitude coffee. Ethiopia is well known for being the home of Arabica coffee and its fine quality coffee, which is acclaimed for its aroma and flavor characteristics. Ethiopia can potentially increase coffee production due to its suitable elevation, climate, and indigenous quality coffee varietals [14].

The main coffee-producing areas in Ethiopia are west and southwest, southern, eastern, and central regions [21]. The main coffee growing or cultivation areas are found within the Oromia Region and Southern Nations, Nationalities, and Peoples’ Region (SNNPR), with modest production in the Amhara Region and minor or limited output in the Benishangul-Gumuz Region. These regions also have specialized and high-value coffee production and cover mainly agroecological zones with the highest agricultural potential area and the main coffee production districts in Ethiopia as well.

## Methods

### Coffee distribution data and sample selection

**Fig 2:**
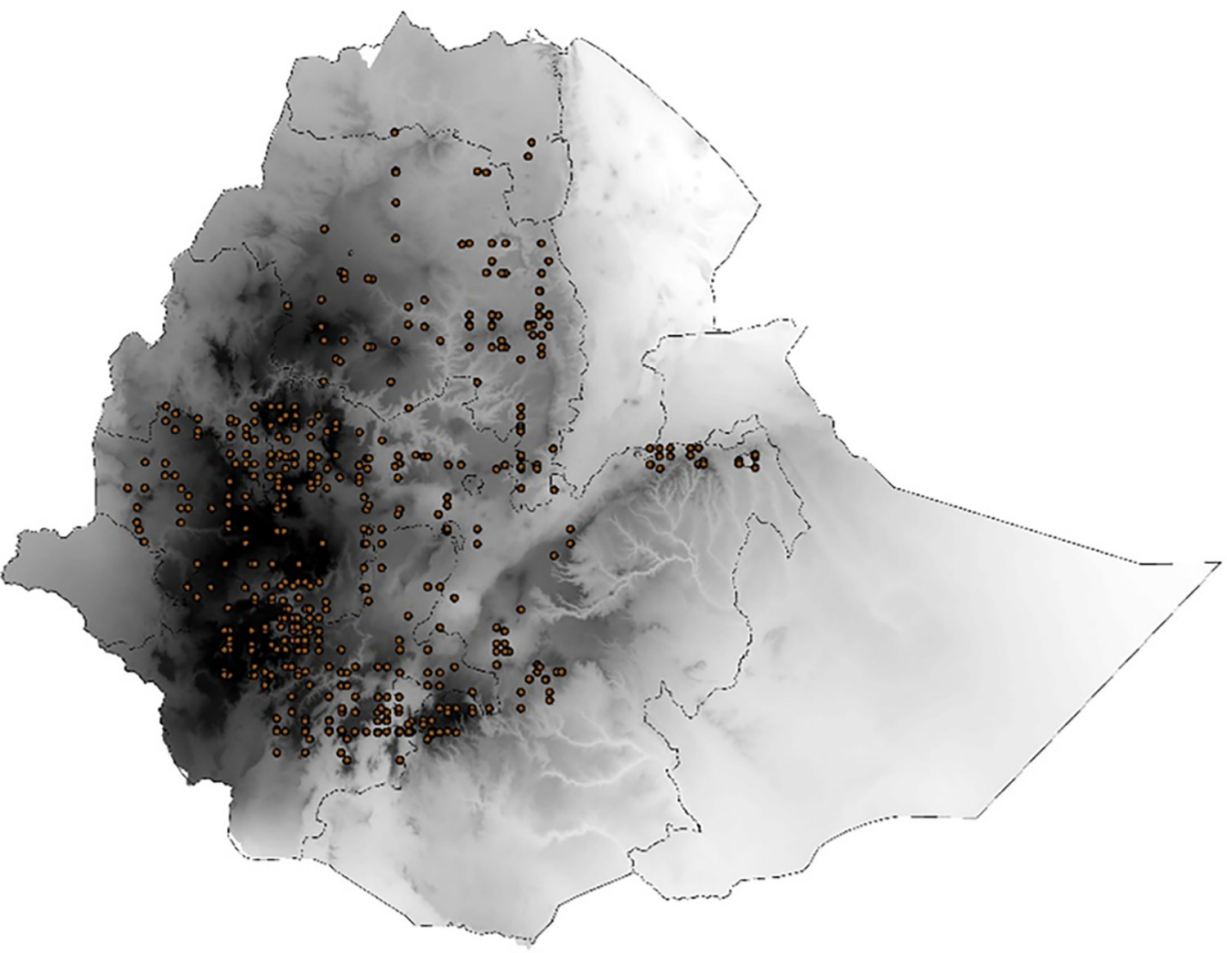
Map of the occurrence points of C.arabica production.

Current climatic suitability data for coffee production was collected using field survey in Ethiopia (Fig 2). Additional locations suitable to produce C.arabica were identified based on information obtained from the Ethiopian Coffee and Tea Development Authority(ECTDA),national coffee research institute of Ethiopia and literature reviews. More than 700 occurrence points known for growing arabica coffee were considered for the study (see suplmentary material). 75% of the C. arabica occurrence data were fitted into the model in association with bioclimatic variables, and the remaining 25% of the data was used to assess model evaluation [22]. The following figure shows the occurrence points of coffee production in Ethiopia.

### Source and selection of environmental variables

Suitability models fitted in present conditions can be transferred to future periods based on emissions scenarios to determine and quantify potential variations in species spatial ranges attributed to climate change [23]. The nineteen bioclimatic variables were obtained from WorldClim database (http://www.worldclim.org) [18] that are CMIP6 downscaled and represent minima, maxima, and seasonality in temperature (°C) and precipitation (mm) at a spatial resolution of 30 arc-seconds (1 km^2^).

The bioclimatic variables were extracted from monthly temperature and rainfall records worldwide.Typically, the variables are utilized in ecological niche modeling in order to generate more biologically meaningful variables [24]. The accuracy of the resulting prediction can be negatively impacted by overfitting of the model due to high correlation and collinearity between bioclimatic variables [25]. To reduce multicollinearity effects, the Pearson correlation coefficient (r) between each variable was computed, and highly correlated variables (r ≥ 0.80) were omitted in the model [26].

From the highly correlated variables, one was chosen to represent the data based on its predictive power (based on jackknife training gain) [21]. After eliminating highly correlated variables, only nine variables were used in the model. These bioclimatic variables were BIO1, BIO5, BIO6, BIO10, BIO11, BIO12, BIO13, BIO15 and BIO16 (Table 1). The Jackknife test was employed to assess the contribution of each bioclimatic variable to best fit the model. The model was iterated 15 times to assess the threshold, while the Bootsrap was set to 25. Input data preparation of environmental layers and presence points was undertaken in ArcGIS 10.8 before they were used in MaxEnt version 3.3.4 [22].

**Table 1:** Selected bioclimatic variables considered in the study.

Code	Description	unit
BIO1	Annual Mean Temperature	°C
BIO5	Max Temperature of Warmest Month	°C
BIO6	Min Temperature of Coldest Month	°C
BIO10	Mean Temperature of Warmest Quarter	°C
BIO11	Mean Temperature of Coldest Quarter	°C
BIO12	Annual Precipitation	mm
BIO13	Precipitation of Wettest Month	mm
BIO15	Precipitation Seasonality (Coefficient of Variation)	percent
BIO16	Precipitation of Wettest Quarter	mm

Two GCMs were used to predict future climate conditions. These were the third Hadley Centre Global Environmental Model (HadGEM2) [27] and the Community Climate System Model version 2 (CCSM2) [28], which were available in the WorldClim database at the time of writing the paper. The models were downscaled global climate models (GCMs) from the Coupled Model Inter-comparison Project Phase 6 (CMIP6).The models were selected due to their (1)exhaustive bias-corrected data for the periods under study (2021–2040, 2041–2060 and 2061–2080). and (2) their better performance in the Ethiopian environment [29–31]. The representation concentration pathway (RCP) 4.5, which is an optimistic scenario shortly after 2100, a lower long-run radiative forcing target level was used in the models [27]. The study acknowledges the difference in the equilibrium climate sensitivity(ECS) between the two models, where CCSM2 and HadGEM2 have ECSs of 5.16 and 5.55, respectively [32]. This difference in climate sensiyivity between the two models would be a merit to this study as it is based on only the optimistic RCP.

### Modelling approach

A ’presence-only’ modeling of environmental niche a was employed to determine C. arabica’s current and future distribution. Presence-only models can generate reliable predictions from a few presence datasets, and it is cheaper option to absence data [26]. Maximum Entropy (MaxEnt) Modeling was employed to assess current and future bioclimatic suitability for coffee production in Ethiopia. MaxEnt v.3.4.4, was download from http://biodiversityinformatics.amnh.org/open_source/maxent/. Maxent predicts species distribution based on the limits of environmental variables for species relative to the background niche availablity [33]. It uses the areas where the species (in this case, C.arabica) is found and associates it with the environmental variables deemed important for predicting occurrences [34]. In MaxEnt, the probability of species occurrence in an area due to environmental variables can be computed as a sum of each weighted variable divided by a scaling constant. This will provide the output as a probability between 0 (not likely to occur) and 1 (most likely to occur) [35].

The nine bioclimatic variables were converted to ASCII raster format using ArcGIS 10.8. The ASCII format data were then imported into the MaxEnt v.3.4.4 along with the species data (with csv format) for suitability prediction [22]. The software package’s basic settings included the following parameters: a random seed, a random test percentage of 25, 10 replicates, and a replicated run type of ‘subsample’. 75% of the distribution points were randomly chosen for the training set and the remaining 25% were used for the test set when the random test percentage was set to 25% [29]. The model was run ten times with the identical parameters by setting the number of replicates to ten. The final result was obtained by averaging the output of each run.

A sample selection bias, which might affect presence-only modeling [36], is expected in such a type of modeling. This is because the sample points would not be uniformly gathered from all parts of the study areas (whereby some parts of the study area might be sampled more frequently than others). However, occurrence data were collected based on the known coffee producing areas without duplication [30]. In this case, MaxEnt removes duplicate presence point by default,i.e points within the same grid cell will be reduced to only one occurrence, thereby reducing spatial biases.

### Model training and validation

MaxEnt has a validation technique that helps assess the modeling output. The most common technique is plotting all sensitivity values (true positive fraction) on the y-axis against their equivalent (1—specificity) values (false positive fraction) for all available thresholds on the x-axis [26]. The technique helps generate the receiver operating curve (ROC), which has been used as as a threshold- independent measure of predictive accuracy [37]. The Area Under the Receiver Operating Characteristics Curve (AUC) was used to assess model performance, which ranges from 0.0 to 1.0 [38]. An AUC value of less or equal to 0.50 represents the lower performance of the model than random distribution, and an AUC value close to 1.0 shows better performance of the model [38,39]. The model performed well if the AUC is greater or equal to 0.78 [40]. Moreover, the True Skill Statistics(TSS), a better measure for the predictve performance of presence–absence predictions than others like kappa, was employed as a threshold- dependent measure in the study. The TSS statistic, provides results that are highly correlated with the threshold-independent AUC statistic [37]. It ranges from −1 to +1, where +1 represents perfect agreement and values of zero or less indicate a performance of the model no better than random.

The sensitivity- specificity sum maximization technique was employed to convert the bioclimatic suitability into different suitability classes [41]. The final probability range (0–1) was generated by the model and then converted into percentage intervals to visualize and aggregate easily in ArcGIS 10.8. Finally, the percentage intervals were transformed into five categories- not suitable N (0–0.2), marginally suitable (S4) (0.2–0.4), moderately suitable (S3) (0.4–0.6), suitable (S2) (0.6–0.8), highly suitable (S1) (0.8–1.0). The area coverage and change in each class were also computed and analyzed [42]. The final ASCII outputs in maps and figures from the MaxEnt were converted into raster format using the Arc Toolbox tool in the ArcGIS software [43].

## Results

### Effect of variables on the ecological niche of C. arabica

**Fig 3:**
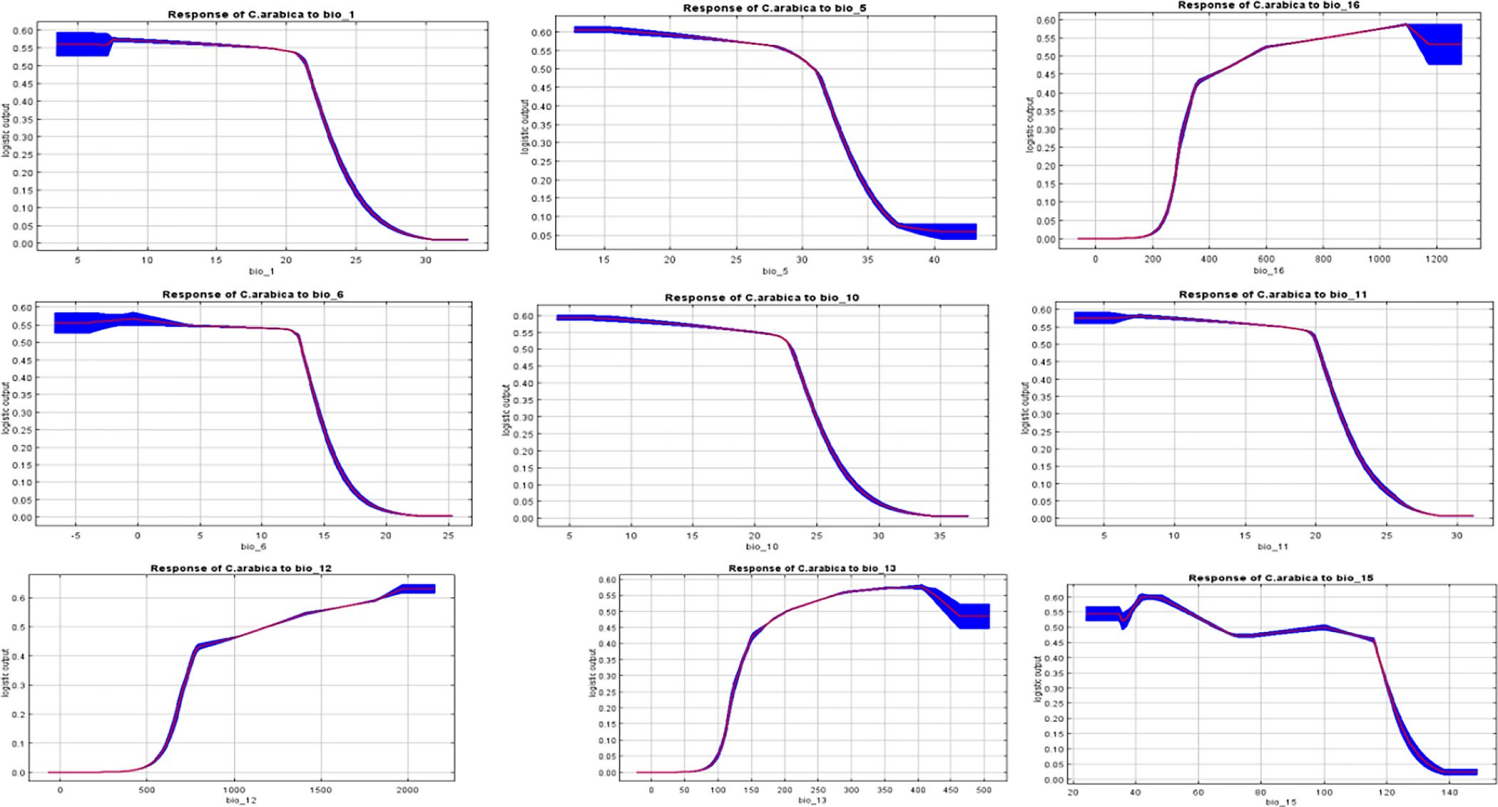
Response curve of the bioclimatic variables.

Fig 3 shows the response curves that depict how the predicted probability change in C. arabica’s presence with the variation of each environmental variable, considering other environmental variables at average sample value. The response curves show how coffee responds to different values of each variable by running a simulation based on only that variable. For example,annual precipitation(BIO12) that affects C. arabica production significantly showed an increasing suitability as the precipitation increased begining from 500mm. In addition,the response curves predict that coffee grows best iin areas with mean annual temperatures between 15°C to 23°C (Fig 3).

Generally, all the temperature variables have been negatively responded to by coffee suitability with dropping off and rising at different points. On the other hand, BIO12’s response curve predicts the mean annual rainfall, showing that optimal coffee production is possible with 500mm and that suitability increases with increasing annual rainfall. Unlike the temperature variables, many of the rainfall variables have been positively responded to the coffee plants; increasing rainfall increases the suitability of the coffee plants.

### Model performance

**Fig 4:**
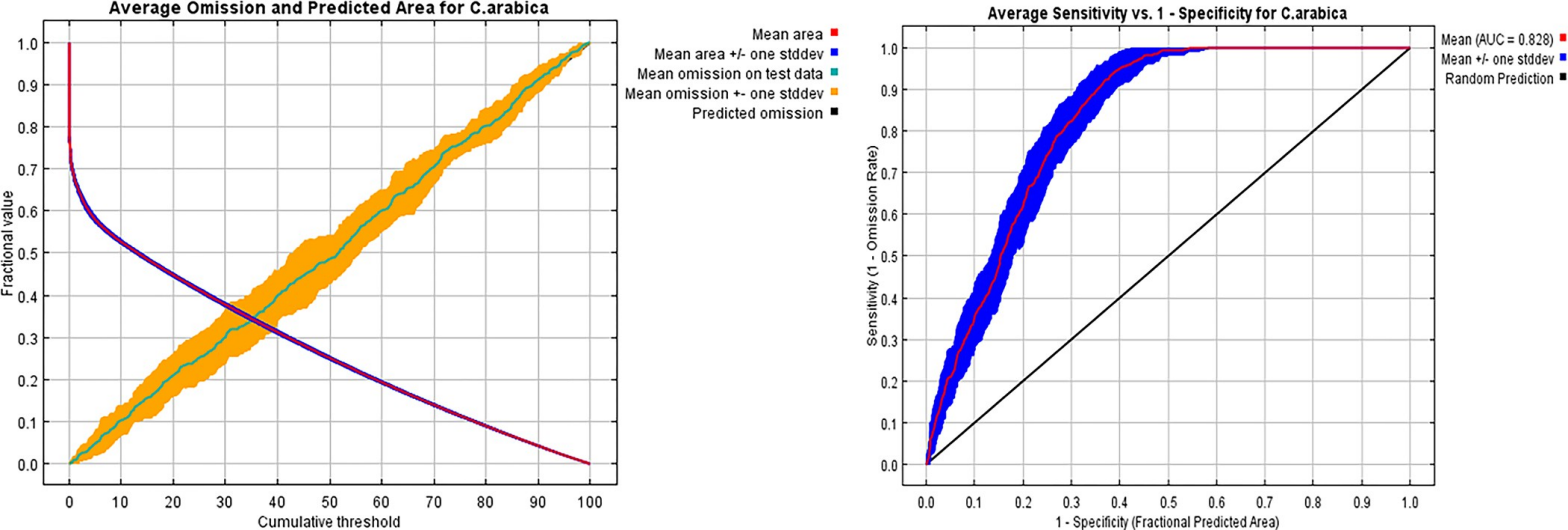
Receiver operating characteristic (ROC) curve for the data.

The AUC value determines whether the model’s prediction of the distribution of C. arabica is better or worse than randomly plotting the distribution of coffee. The model of the coffee distribution indicated an AUC value of 0.82 with a standared deviation of 0.02, indicating the simulated model performed well and can be used for adequate analysis (Fig 4). Under the current climatic conditions, a visual assessment of the prediction about the presence points revealed a consistent agreement between the presence points and predicted coffee suitability.The true skill statistic(TSS) value(0.89) also indicates a better performance of the presence-absence predictions(36).

### Variable importance analysis

Table 2 indicates the estimates of the relative contributions of the climatic variables to the Maxent model. Precipitation and temperature-related variables were important in determining suitability for coffee production in Ethiopia. The annual rainfall (BIO12), which accounts for 54.3% of the expected change in suitability, is the most important climatic variable. The month of the calendar year when Ethiopian coffee cherries ripen and are harvested, BIO11(mean temperature of October,November and December for Ethiopia), has a mean temperature that contributes roughly 16.1% of the total. This implies that the plant experiences physiological stress as a result of the berry ripening and is unable to withstand extra stress brought on by high temperatures and a lack of water. The warmest quarter’s(April,June and July for Ethiopia) mean temperature (BIO10) contributed 10.1%, the wettest quarter’s(June July and August fro Ethiopia) precipitation (BIO16) contributed 5.2%, and the annual mean temperature (BIO1) contributed 10.5%. These results emphasize the significance of adaptation strategies to lessen the effects of variables related to both temperature and rainfall.

**Table 2:** Contribution of the different bioclimatic variables to the predicted change in climatic suitability for C. arabica production in Ethiopia.

Variable	Present mean	Change by 2080	Percent contribution	Permutation importance
BIO12	2750	-123	54.3	27.4
BIO11	16.3	+2.2	16.1	22.3
BIO5	28.8	+1.6	1.7	7.2
BIO15	758.8	-24.9	1.2	15.3
BIO10	23.4	+1.1	10.1	0.1
BIO16	1080.2	-98	5.2	7.5
BIO13	250.7	-19	0.5	4.5
BIO1	20.3	+1.19	10.5	9.7
BIO6	0.23	+2.3	0.4	6.1

On the other hand, the minimum temperature of the coldest month(December for Ethiopia) (BIO6) and precipitation of the wettest month(August for Ethiopia) (BIO13) were less important for change in coffee suitability in Ethiopia. The remaining bioclimatic variables have relatively lower influences in determining the fundamental niche of C. arabica. BIO12 (27.4%), followed closely by BIO11(22.3%), is also by far the most significant variable in terms of permutation importance. Permutation importance is a superior way to indicate the relative importance of each variable because it is based on the final model.

Studies pointed out that the factors that affect the suitability of coffee, in general, vary with geographical scale and typicality. Temperature and precipitation factors are almost equal in determining coffee’s bioclimatic suitability in Ethiopia [23], confirming findings from other studies [44]. However, in global-level studies, temperature factors were identified as the primary determinant factors of Arabica coffee suitability [45]. On the other hand, some national research elsewhere identified precipitation-based factors are more important than temperature in determining suitability [17]. Such variations are explained by differences in scale and geography, respectively, indicating that the potential for coffee can be influenced by local and regional factors [23].

**Fig 5:**
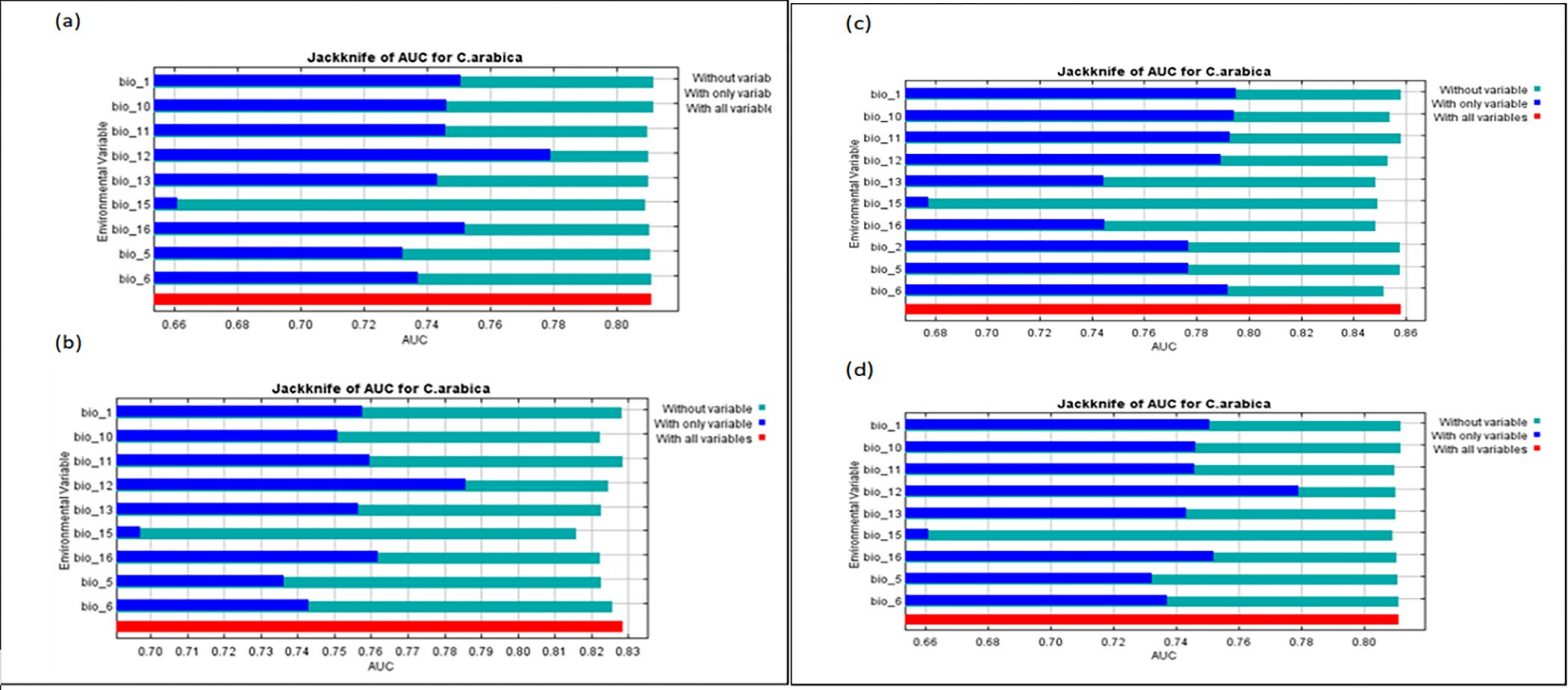
Jackknife on area under curve (AUC) for 2040 under CCSM2 (a) and HadGEM2(b) model; for 2080 under CCSM2(c) and HadGEM2 model (d).

Fig 5 shows the importance of each variable in the model. The importance is determined through how much ’gain’ over a random distribution the variable has and is visualized in the jackknife test for test gain. The most essential variable under ’only variable’ is BIO12 from 2023 to 2040 in both models. On the other hand, BIO1 has a more significant contribution for 2080 in the CCSM2 model.

### Bioclimatic suitability for C. arabica under current and future climate

#### Period 1: Coffee suitability from 2021–2040

**Fig 6:**
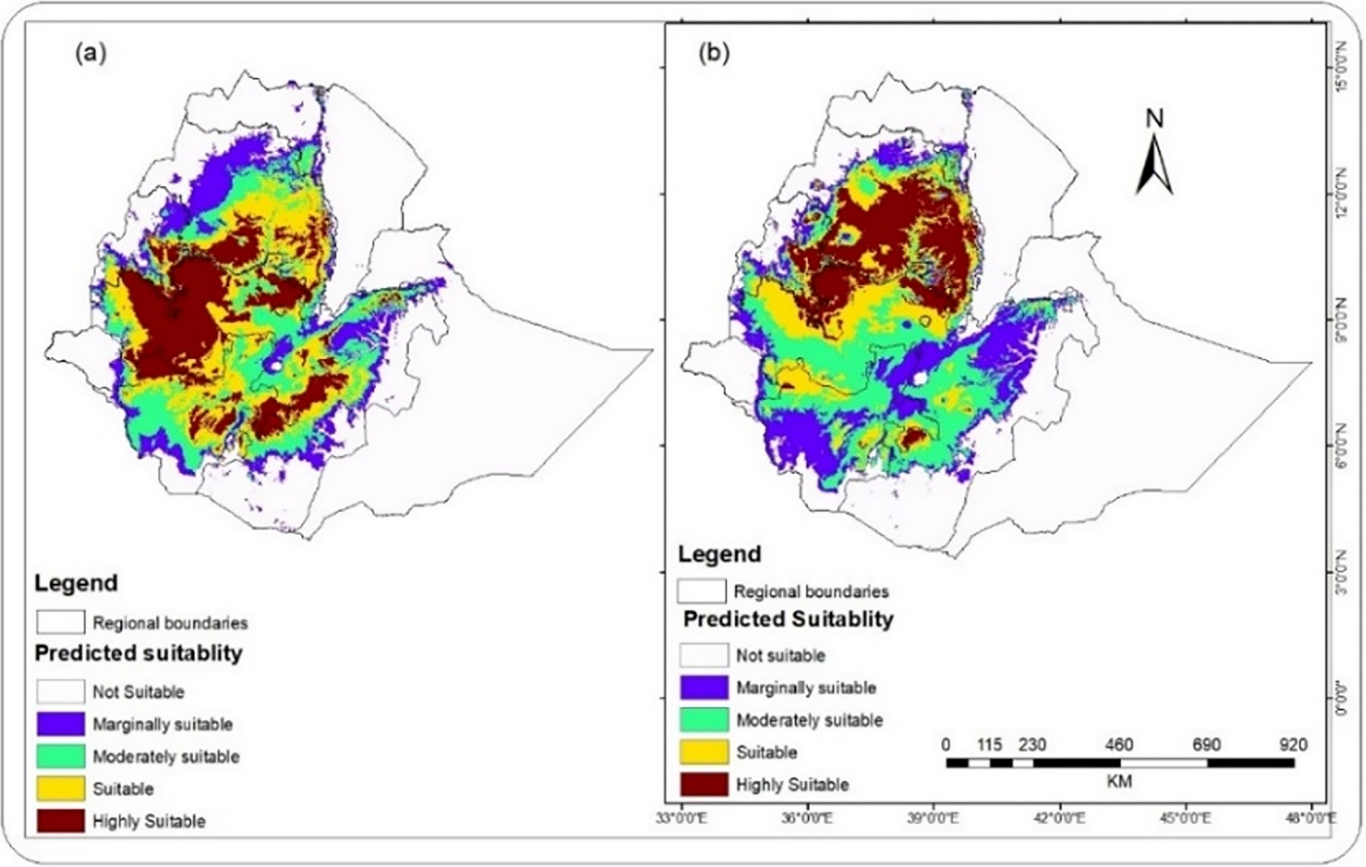
Current climate suitability for arabica coffee based on CCSM2 (a) and HadGEM2 (b) models (The shapefile is extracted from https://hub.arcgis.com/datasets/esri::world-countries-division/explore?location=-1.440657%2C0.000000%2C3.00).

Fig 6 shows the overall current coffee suitability (S1 & S2) in southwest and Southern Ethiopia. The southwestern and southern highlands of the country are highly suitable for C. arabica. However, coffee-growing areas in southeastern and southern Ethiopia will be the most susceptible to future climate change.

As can be seen from Table 3, about 31% and 28% of the country’s area appears to be suitable for coffee production based on the CCSM2 and HadGEM2 models, respectively. By implementing an ensemble model of three machine learning algorithms, a study by [37] found that about 27% of Ethiopia’s area is climatically suitable for coffee production by 2040. The marginally suitable classe(S4) will decrease by more than 10% and 8% based on CCSM2 and HadGEM2 models, respectively, between 2021 and 2040.

**Table 3:** Current (2021) and future (2040) suitability based on CCSM2 and HadGEM2 models.

Suitability Class	CCSM2		Change in %	HadGEM2		Change in %
	2021	2040		2021	2040	
	Area (km^2^)	Area (km^2^)		Area (km^2^)	Area (km^2^)	
S1	125,879.78	132,379.37	5.16	114,136.02	121,654.94	6.59
S2	106,596.20	129,228.78	21.23	96,596.20	111,086.53	15.00
S3	31,687.49	45,692.25	44.2	21,687.49	29,449.61	35.8
S4	78,697.76	70,597.27	-10.30	78,697.76	71,948.01	-8.57
Overall	342,860.23	377,897.67	10.19	311,116.47	334,139.09	7.4
% of area	31.00	34.16	3.16	28.13	30.21	2.08
N	763,139.77	728,102.32	-4.59	794,883.53	771,860.91	2.90
Grand Total	1,106,000	1,106,000		1,106,000	1,106,000	-

Note: S1: Highly suitable, S2: Suitable S3: Moderately suitable, S4: Marginally suitable, N: Unsuitable.

On the other hand, suitability classes ranging from S1 to S3 revealed expected increments in coverage to a different extent. For example, the moderately suitable class(S3) is expected to increase to the greatest extent, i.e., 44.20 and 35.80% based on the CCSM2 and HadGEM2 models, respectively. The suitable class(S2) also shows change in 23.23% and 15.00% expansion in CCSM2 and HadGEM2 models, respectively. The highly suitable class(S1), however, will be increased to the lowest extent in both models, which is 5.16% and 6.59% in terms of the CCSM2 and HadGEM2 models by 2040, respectively.

While the different classes of bioclimatic suitability for arabica coffee production indicated varied extent and direction of change by 2040, the overall bioclimatic suitability in Ethiopia depicted an increasing trend based on both models, which is 10.9% and 7.4% for CCSM2 and HadGEM2, respectively. This implies that there are some areas where one class would be converted from one class of suitability to another class of bioclimatic suitability. The decrease(8.57%) in the marginally suitable class(S4) highlights that the marginally suitable areas at a lower altitudinal range of coffee production could be converted to unsuitable regions. On the other hand, there is a decrease in unsuitable class(N) in both models, which is 4.59% and 2.90% for CCSM2 and HadGEM2, respectively. This might be due to the conversion of areas above the higher altitudinal limit to the different suitability classes. This conversion pattern implies an upward shift in bioclimatic suitability for coffee production, which reaffirms the study’s findings by [14] stating that coffee production is moving upward along the different altitudinal zones of the eastern African coffee-producing countries due to climate change.

#### Period 2: Predicted changes in coffee suitability from 2041 to 2060

**Fig 7:**
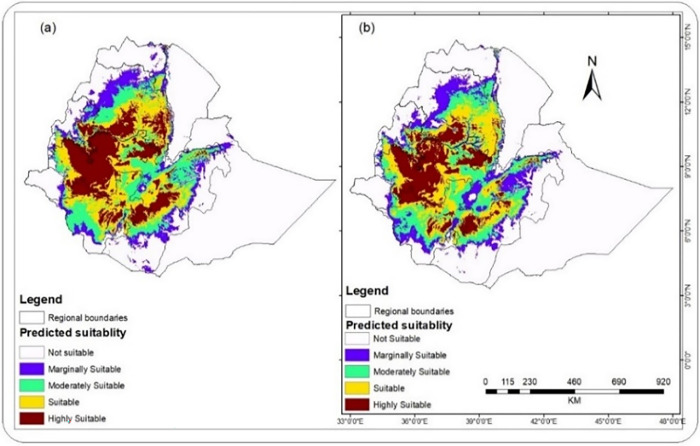
Predicted climate suitability of arabica coffee (2041–2060) based on CCSM2 (a) and HadGEM2 (b) (The shapefile is extracted from https://hub.arcgis.com/datasets/esri::world-countries-division/explore?location=-1.440657%2C0.000000%2C3.00).

Most current growing areas are expected to increase in terms of the different suitability classes while decreasing in terms of area coverage of the unsuitable regions in this period (Fig 7). On the other hand, suitable areas identified before 2040 in southeast and southern Ethiopia are predicted to no longer be suitable habitats for the survival of Arabica coffee. Ethiopia’s eastern and southeastern coffee-producing areas will be most affected by the changing climate, and the suitable regions will shrink. In contrast, areas in southwestern and northcentral Ethiopia will benefit from climate change; future changes in the suitability zones will alter the geographical distribution of potential optimal sites, increasing the suitability and productivity of these areas.

Fig 7 also shows a shift in suitability classes to the west, southwest, and north central parts of Ethiopia, and the habitat is becoming fragmented in the Ethiopian plateau. This indicates that the impact of climate change on coffee production varies spatially and temporally, highlighting the differential vulnerability of systems and exposure units. In general, the area of favorable climatic conditions for growing coffee in Ethiopia will likely be increased, unlike other regions of coffee production in the world, like South and Central American countries whose bioclimatic suitability will shrink. Similar studies conducted for coffee and other species in Ethiopia and globally [16,44] also note a future habitat range reduction and shifts due to the impact of climate change.

Table 4 indicates that the area of all suitability classes (except S4) is predicted to increase in different percentages under both CCSM2 and HadGEM2 models by 2060. Significant increases are expected under the CCSM2 model, i.e., about 25.85%, 21.47%, and 14.10% for the moderate suitable, suitable, and highly suitable classes, respectively. Moreover, the overall climate suitability for arabica coffee in Ethiopia will increase by more than 12.06% and 11.31% under CCSM2 and HadGEM2 models, respectively, during this period (2041–2060). Furthermore, Table 5 depicted that there will be an increase in the percentage of the over suitable area of the country by 4.07% and 3.42% based on CCSM2 and HadGEM2 in this period (2041–2060). This indicates that Ethiopia will benefit from climate change regarding climatic suitability for coffee production. In other words, there are some areas where one class of suitability will be changed to another class of bioclimatic suitability.

**Table 4:** Bioclimatic suitability in (2041) and (2060) based on CCSM2 and HadGEM2 models.

Suitability Class	CCSM2		Change in %	HadGEM2		Change in %
	2040	2060		2040	2060	
	Area (km^2^)	Area (km^2^)		Area (km^2^)	Area (km^2^)	
S1	132,379.37	151,047.85	14.10	121,654.94	139,528.08	14.69
S2	129,228.78	156,985.08	21.47	111,086.53	124,136.02	11.74
S3	45,692.25	57,503.84	25.85	29,449.61	26,596.2	-9.68
S4	70,597.27	57,946.23	-17.92	71,948.01	81,687.49	13.53
Overall	**377,897.67**	**423,483.00**	**12.06**	**334,139.09**	**371,947.8**	**11.31**
% of area	34.16	38.23	4.07	30.21	33.63	**3.42**
N	728,102.32	682,517.00	**-**10.38	771,860.91	734,052.2	-4.89
Total	1,106,000	1,106,000		1,106,000	1,106,000	

Note: S1: Highly suitable, S2: Suitable S3: Moderately suitable, S4: Marginally suitable, N: Unsuitable.

**Table 5:** Coffee production suitability in 2060 and 2080 based on CCSM2 and HadGEM2.

Suitability Class	CCSM2		Change in %	HadGEM2		Change in %
	2061	2080		2061	2080	
	Area (km^2^)	Area (km^2^)		Area (km^2^)	Area (km^2^)	
S1	132,379.37	166,056.68	25.44	139,528.08	171,759.07	23.10
S2	129,228.78	185,753.45	43.74	124,136.02	172,549.07	39.00
S3	45,692.25	35,630.82	-22.02	26,596.2	19,415.23	-27.00
S4	70,597.27	61,426.68	-12.99	81,687.49	69,172.97	-15.32
overall	**377,897.67**	**447,015.15**	**18.29**	**371,947.8**	**448,717.83**	**20.64**
% of area	34.16	40.41	6.25	33.63	40.57	6.94
N	728,102.33	658,984.85	-9.49	734,052.2	657,282.17	-10.45
Total	1,106,000	1,106,000		1,106,000	1,106,000	

**Note:** S1: Highly suitable, S2: Suitable S3: Moderately suitable, S4: Marginally suitable, N: Unsuitable.

The marginally suitable class, on the other hand, is predicted to decrease by 17.92% under the CCSM2 model. The HadGEM2 model predicts that the moderately suitable class(S3) will be decreased by 9.68% in 2060. In contrast, the suitability (S1), suitable(S2) and the marginally suitable(S4) classes will be increased by 14.69%, 11.74% and 13.5%, respectively. The change of S3 and S4 in opposite direction under the two models might be due to variation in the equilbrium climate sensitivity of the two models. Moreover,the decrease in the marginally suitable class(S4) in CCSM2 and moderately suitable class(S3) indicates that areas at lower altitudes of coffee production could be converted to unsuitable areas. On the other hand, there is decrease in unsuitable class(N) in 10.38% and 4.89% for CCSM2 and HadGEM2, respectively. The reduction might be due to the conversion of areas above the higher altitudinal limit to the different suitability classes. This suggests an upward shift in bioclimatic suitability for coffee production, which corroborates with the study’s findings by [14] stating that coffee production is moving upward along the different altitudinal zones of the eastern African coffee-producing countries due to climate change.

#### Period 3: Predicted changes in coffee suitability from 2061 to 2080

**Fig 8:**
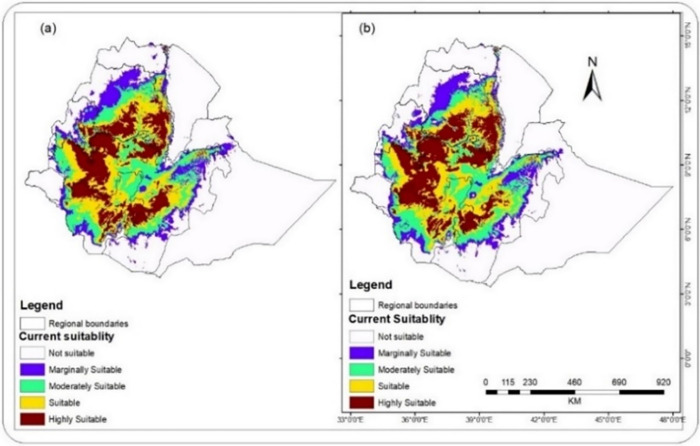
Predicted climate suitability for C. arabica in 2061–2080 based on CCSM2 (a) and based on HadGEM2 (b) (The shapefile is extracted from https://hub.arcgis.com/datasets/esri::world-countries-division/explore?location=-1.440657%2C0.000000%2C3.00).

Many studies note that the mean annual temperature has been projected to go up 1.1–3.1°C and 1.5–5.1°C by 2060 and 2090, respectively, in Ethiopia [46], albeit there are marked microclimatic variations. The predicted increase in temperature will have unprecedented impacts on coffee production worldwide, particularly on the loss of suitable land for coffee production. Fig 8 shows that the main coffee-producing areas investigated in Ethiopia (east, south, and southeast coffee-producing regions of Ethiopia) would seriously be affected by climate change with a substantial decline in suitable places.

Table 5 also shows that the area of all suitability classes is predicted to be changed in different percentages under both CCSM2 and HadGEM2 models by 2080. During this period, in both models, there will be substantial decrease in moderately suitable (S3) and marginally suitable (S4) classes. The moderately suitable(S3) class will shrink from 45,692.25 km^2^ in 2060 to 35,630.82 km2 in 2080- which is a decrease of 22.02% in this period under the CCSM2 model. Besides, the same model predicts that this class will be decreased from 26,596.2 km^2^ in 2060 to 19,415.23 km^2^ in 2080; which indicates a decrease of 27.00% in this period.

Moreover, a remarkable decrease in the coverage of the marginally suitable (S4) class will be observed based on the CCSM2 model. This class will be diminished from 70,597.27km^2^ to 61,426.68km^2^ in this period (2061–2080), which represents a decrease of 12.99% under the CCSM2 model. The HadGEM2 model also predicts that the coverage of the S4 suitability class will decrease from 81,687.49km^2^ in 2060 to 69,172.97km^2^ in 2080, which reveals a decrease of 15.32% in this study period. The reduction in both classes of bioclimatic suitability under the two models may correspond to the scenario that climatic suitability for coffee production shrinks at lower altitudes in many coffee-producing regions of the world.

In contrast, the highly suitable(S1) class will be expanded from 132,379.37 km^2^ in 2060 to 166,056.68 km^2^ in 2080, an increase of 25.44% under the CCSM2 model. Similarly, this class revealed a change from 139,528.08 km^2^ to 171,759.07 km^2,^ which represents an increase of 23.10% under the HadGEM2 model. Moreover, a marked rise in the coverage-of suitable (S2) class will be observed based on the CCSM2 model. This class will be changed from 129,228.78km^2^ to 185,753.45km^2^ in this period (2061–2080), representing an increase of 43.74% under the CCSM2 model. Furthermore, the HadGEM2 model predicts that the coverage of the S2 suitability class will be increased from 124,136.02 km^2^ to 172,549.07 km^2,^ which represents an increase of 39.00% in this study period. The increase in both classes of bioclimatic suitability under the two models can be attributed to the upward shift of climatic suitability toward higher altitudes in many coffee-producing regions of the world. This reaffirms the finding of [12], which indicated the expansion of coffee cover in Ethiopia, evidently stating that coffee yield decreased while production increased in the last three decades.

While the different classes of bioclimatic suitability for arabica coffee production indicated varied extent and direction of change by 2080, the overall bioclimatic suitability in Ethiopia depicted an increasing trend based on both models, which is 18.29% and 20.64% for CCSM2 and HadGEM2, respectively, between 2061 and 2080. This suggests some areas with one suitability class will be converted to another class of bioclimatic suitability. The decrease in the marginally suitable class(S4) highlights that the marginally suitable areas in lower altitudinal parts of coffee production could be converted to unsuitable regions.

On the other hand, there is a decrease in unsuitable class(N) in both models, which is 9.49% and 10.45% for CCSM2 and HadGEM2, respectively. This decrease might be due to the expected conversion of areas in the higher altitudinal limit to the different suitability classes. The upward shift of both the lower and upper limits of the altitudinal range for coffee production, in turn, implies that there is an upward shift in bioclimatic suitability for coffee production, which reaffirms the findings of the study by [14] stating that coffee production is moving upward along the different altitudinal zones of the eastern Africa coffee producing countries due to climate change. The results from a study by [44] also show that the area suitable for coffee in Ethiopia will increase gradually until the 2090s.

**Fig 9:**
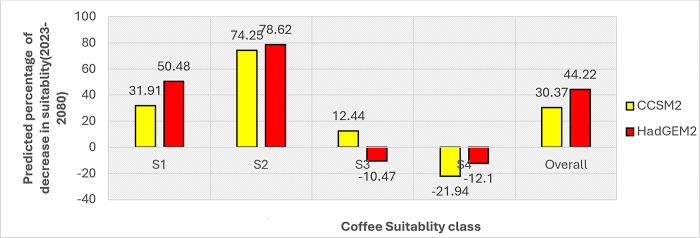
Predicted change in coffee suitability in 2080 based on the two models.

Fig 9 depicts the percentage of change in the coverage of the different suitability and the overall suitability over the study period (2021–280) under the two models. As shown from the figure, the area of suitability classes is predicted to be impacted significantly under both CCSM2 and HadGEM2 models by 2080. Substantial increases are expected in the highly suitable class under both models: 31.91% and 50.48% under the CCSM2 and HadGEM2 models, respectively. The climatically suitable class also shows a drastic increase in both models, which is 74.25% and 78.62% for the CCSM2 and the HadGEM2 models, respectively. Unexpectedly, the two models predicted the moderately suitable class in the opposite direction: an increase by the CCSM2 and a decrease by the HadGEM2. In contrast, both models indicated a significant decrease in the marginally suitable area. A decrease in the marginally suitable class is expected with 21.94% and 12.1% under the CCSM2 and HadGEm2 models, respectively.

Under the two models, the overall bioclimatic suitability for coffee production will be increased by 2080. It will be increased by 30.37% and 44.22% under the CCSM2 and HadGem2 models, respectively. The overall increase in suitability under the two models corresponds to the finding from a study by [9], which indicated the expansion of coffee cover in Ethiopia, evidently stating that coffee yield decreased. At the same time, production increased in the last three decades.This finding aligns with other studies on climate change impacts on coffee in the country, which show that suitability will increase [10,12]. There is a general pattern of increase in the area suitable for coffee production in Ethiopia.

## Discussion

The study highlights that even with limited, sparse, and irregularly sampled data, the MaxEnt model was able to produce reliable findings, which are visualized using ArcGIS. In fact, for many species, the sample field data are insufficient to accurately describe the geographic distribution of the species(C.arabica in this study), and digital specimen information is also frequently needed. However, due to a lack of precise latitude and longitude data, the distribution records of some species need to be verified by using other ways like field observation and Google Earth [46]. Climate factors are the only environmental variables utilized in the MaxEnt model in this study. Nine bioclimatic variables—temperature and precipitation related—were selected depending on their potential effect on the distribution C. arabica and the multicollinearity that exist between them. However, the distribution of coffee production is influenced by various factors, including political economy, market forces, and climate. Consequently, the MaxEnt model illustrates the theoretical maximum probable distribution of species, often showing areas much larger than those inhabited. Using the models and n the principle of maximum entropy to predict coffee distributions, and then generating knowledge about bioclimatic suitability that would be an input to the efforts in building sustainable coffee livelihood.

The study reveals that variations in temperature-related variables, such as yearly temperature (BIO1) and annual precipitation affect coffee suitability. In particular, annual precipitation (BIO12) has a greater impact on future changes in the appropriateness of coffee production. These findings extend that of Chemura et al [12] reaffirming that coffee suitability is highly influenced by precipitation amount and distribution.This highlights that both precipitation and temperature in Ethiopia will likely determine the future bioclimatic suitability of arabica coffee. The study highlights that there will be an overall change in the suitability of coffee-growing areas both in space and time within Ethiopia in the next decades. These changes will be mainly positive regarding the predicted climatic suitability for coffee production, although some suitability classes are expected to be negatively affected under the two models in the next decades. This means that many areas that are unsuitable for coffee growing in the present time will become suitable in the future. In some cases, others will be unsuitable in Ethiopia. Most notably, important coffee-producing areas, including southeast and eastern coffee-producing regions of the country, will suffer the most significant decrease in suitability, and the west, southwest, and central highlands will show a substantial increase in suitability. Some suitability classes also show change in opposite directions under the two models. Such irregularities might attribute to the difference in the equilibrium cimate sensitivity of the two models [37].

This study also divulged two different phenomena about the predicted climatic suitability for arabica coffee production. First, it has been identified that Ethiopia’s overall suitable coffee areas will increase (as indicated by different suitability classes) in the next decades, indicating Ethiopia will benefit from future climate change in terms of bioclimatic suitability. Huge areas of unsuitable areas, particularly at higher altitudes, will be suitable under the two models by 2080. Moreover, the findings have shown that climate change will have contribution in suitability of areas, as indicated by change in the area of the different classes of suitability. New areas for coffee production will be opened in Ethiopia, particularly in the country’s western, central, and north-central parts. This finding generally agrees with the findings of [9,15,47], which stated that the spatial range of coffee production in Ethiopia will be moved up to higher altitudes in response to the changing climate. Moving up these areas will ensure resilience in the Ethiopian coffee sector [48,49]. However, the change in the spatial range of coffee production due to climate change will increase the pressure on land in the new coffee-producing areas[23]. This shift requires short and long term adaptation strategies for Ethiopia’s sustainable and more climate-resilient coffee production system.

Finally, the main limitation of this work is that it does not include other impacts of climate change on coffee, including pests, diseases, weeds, and pathogens. Future impacts of pests and diseases, attribute to climate change, on coffee are projected to be more damaging to Ethiopia’s coffee yield, quality, and production [50,51]. Farmers in many areas of Ethiopia reported coffee yield losses due to pests’ increased expenses in control of the optimal output. Besides, the impacts of climate change are projected to cause a decrease in the yields of coffee due to diseases and weed outbreaks [52]. Many other important factors drive change in the spatial range of coffee production, such as markets, social and cultural preferences, and policies that would have been incorporated into this study. Such analyses are not included in the current analysis and remain understudied.

## Conclusions

The study came up with three major findings: an increase in overall suitability for coffee production in new areas of Ethiopia, change in the areas of the different classes of suitability under the two models, and an apparent shift in suitability for coffee production in the southwest and central highlands of Ethiopia, particularly by 2060 and later. Furthermore, although it is beyond the scope of this study, and research is needed to fully understand the multiple drivers of change in the spatial range of coffee areas, it appears clear that changes in the political economy of coffee sector influence coffee production.

The results of this study have practical implications in Ethiopia and beyond. Some of the implications might be building a more climate-resilient coffee production system in currently suitable areas. How can coffee production be sustainable and climate resilient in areas suitable for coffee production? Agronomic management may be modified in regions that will still be good for coffee production but will no longer be as suited to mitigate the effects of climate change; for example, drought-resistant varieties and shade cover are all useful practices that can be implemented. Moreover, designing strategies to transform into a coffee livelihood in newly suitable areas is important. However, further studies that consider the political economy of coffee are needed to substantiate whether coffee production can be undertaken in potentially suitable areas where it is not currently observed. It is necessary to conduct explicit research on the future distribution of climatically favorable places for coffee production, especially at local scales, in order to identify and evaluate any potential conflicts and trade-offs with current land uses. Furthermore, how can coffee farmers be helped in areas where they cannot shift to other livelihoods? This is another practical implication. Moving on to other livelihoods in areas that will be unsuitable, we will need to identify alternative livelihoods in future climate scenarios. These practical implications are in the political economy’s framework and the stakeholders’ power relations in Ethiopia’s coffee sector and beyond.

The study is based on two GCMs and SSPs4.5. The difference in increase of suitable areas for coffee production under the two models would seem attributed to difference in equilibrium climate sensitivity between the two modelsThis difference might have implications on the results about impacts of climate change and opens other area for further research on the effects of ECS on the predictive performance of the models.Thus, it is recommended that further study be conducted on the predicted impact of climate change on C. arabica in Ethiopia based on a greater number of GCMs and all four shared socioeconomic pathways and future periods. Future works should also include climate change driven diseases and pests as well as the political economy aspects of climate change.

## Supporting information

S1 File (CSV)
